# Supplementary material for: Phytosome-Encapsulated 6-Gingerol- and 6-Shogaol-Enriched Extracts from Zingiber officinale Roscoe Protect Against Oxidative Stress-Induced Neurotoxicity
Source: Molecules. 2024 Dec 22;29(24):6046. doi: 10.3390/molecules29246046 (PMC11677370; doi:10.3390/molecules29246046)
Supplement: Supplementary file 1 [file molecules-29-06046-s001.zip › molecules-3356683-supplementary.pdf]

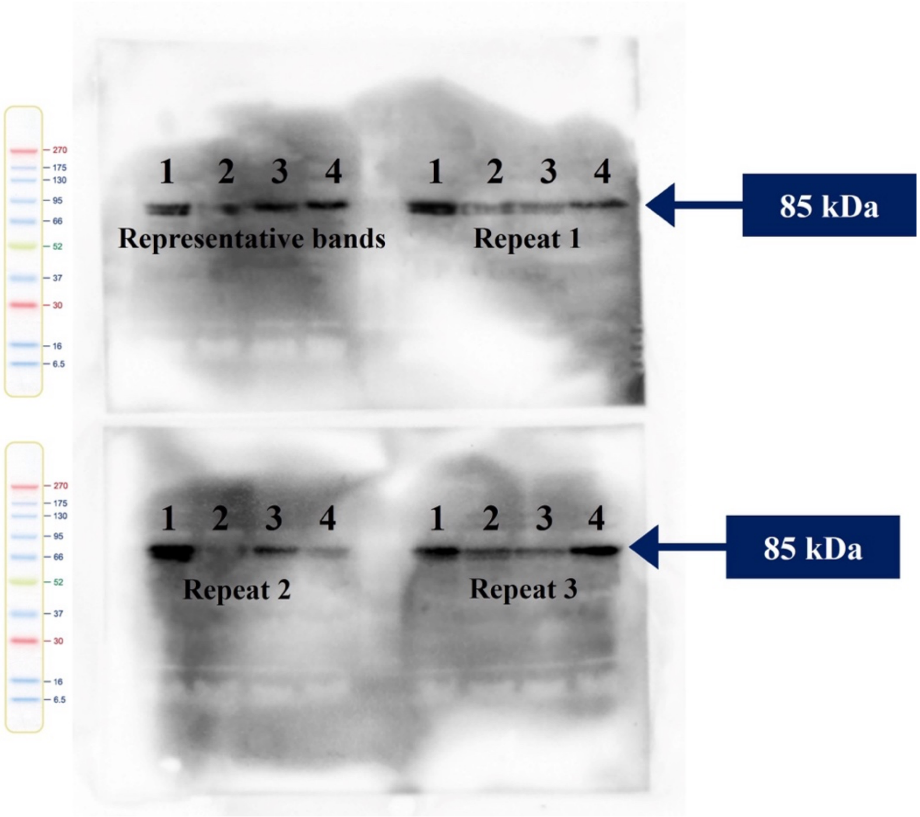

Figure S1: Western blotting was used to detect the expression of PI3K in response to the effects of phytosome-encapsulated 6-gingerol and 6-shogaol-enriched extracts from *Zingiber officinale* Roscoe (6GS) on hydrogen peroxide-induced neurotoxicity in SH-SY5Y cells. The groups included: (1) control (2) hydrogen peroxide + vehicle, (3) hydrogen peroxide + 6GS at a dose of 15.625 µg/mL, and (4) hydrogen peroxide + 6GS at a dose of 31.25 µg/mL.

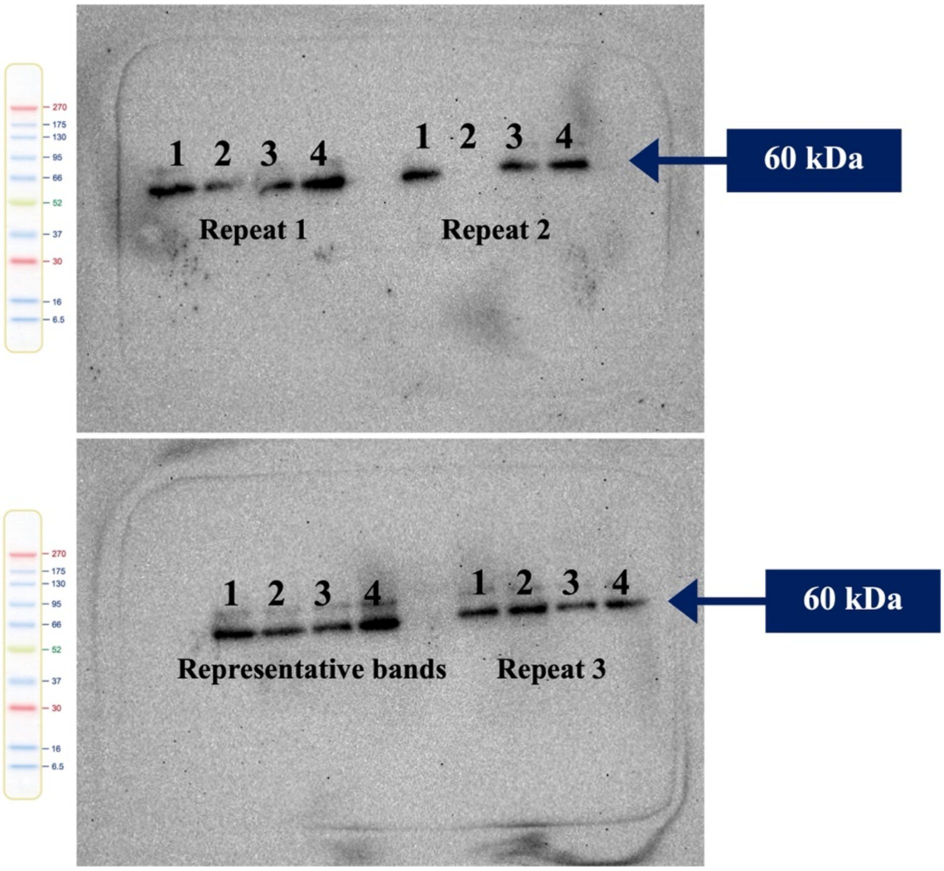

Figure S2: Western blotting was used to detect the expression of p-Akt in response to the effects of phytosome-encapsulated 6-gingerol and 6-shogaol-enriched extracts from *Zingiber officinale* Roscoe (6GS) on hydrogen peroxide-induced neurotoxicity in SH-SY5Y cells. The groups included: (1) control, (2) hydrogen peroxide + vehicle, (3) hydrogen peroxide + 6GS at a dose of 15.625 µg/mL, and (4) hydrogen peroxide + 6GS at a dose of 31.25 µg/mL.

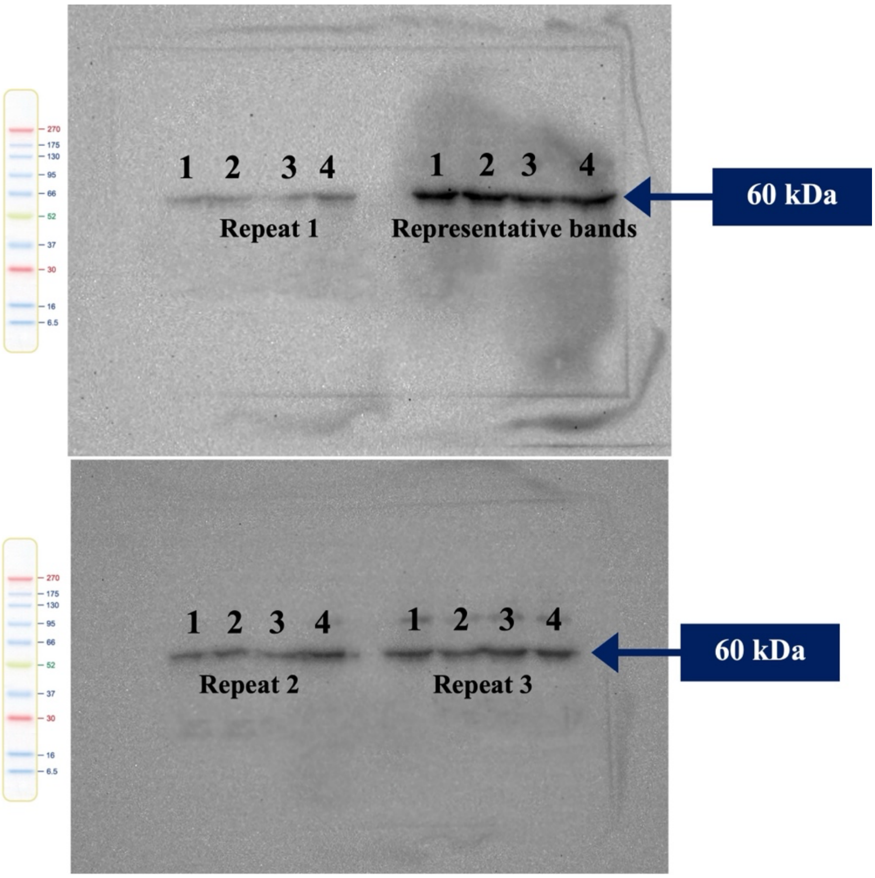

Figure S3: Western blotting was used to detect the expression of Akt in response to the effects of phytosome-encapsulated 6-gingerol and 6-shogaol-enriched extracts from *Zingiber officinale* Roscoe (6GS) on hydrogen peroxide-induced neurotoxicity in SH-SY5Y cells. The groups included: (1) control, (2) hydrogen peroxide + vehicle, (3) hydrogen peroxide + 6GS at a dose of 15.625 µg/mL, and (4) hydrogen peroxide + 6GS at a dose of 31.25 µg/mL.

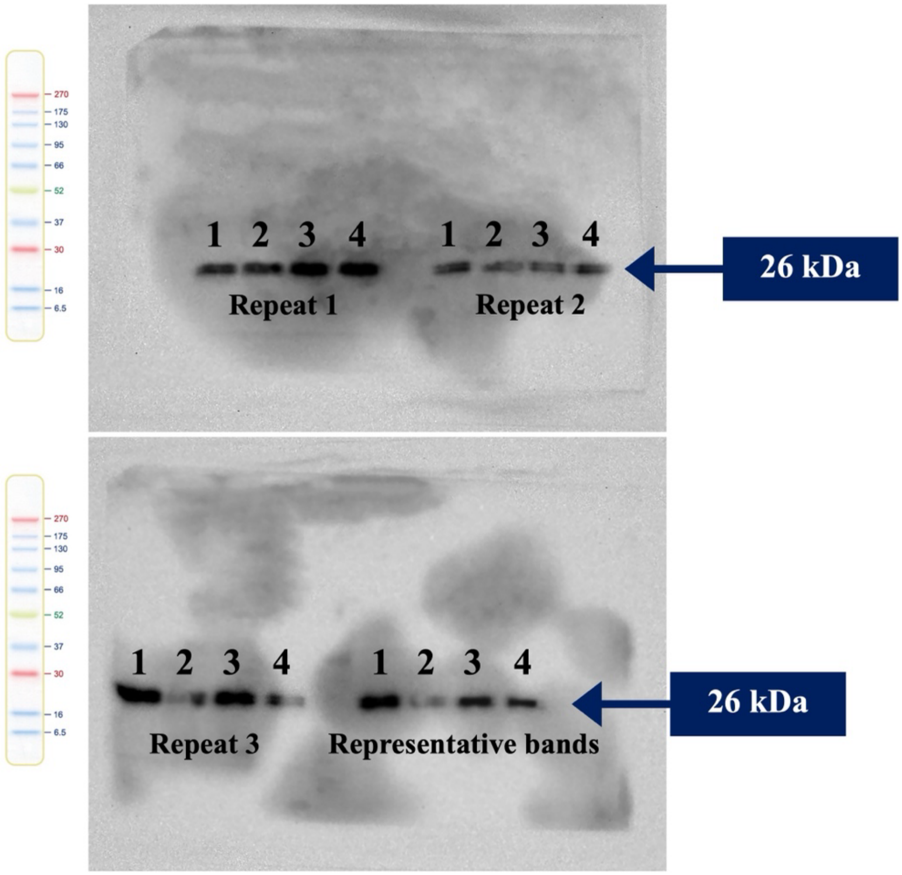

Figure S4: Western blotting was used to detect the expression of Bcl-2 in response to the effects of phytosome-encapsulated 6-gingerol and 6-shogaol-enriched extracts from *Zingiber officinale* Roscoe (6GS) on hydrogen peroxide-induced neurotoxicity in SH-SY5Y cells. The groups included: (1) control, (2) hydrogen peroxide + vehicle, (3) hydrogen peroxide + 6GS at a dose of 15.625 µg/mL, and (4) hydrogen peroxide + 6GS at a dose of 31.25 µg/mL.

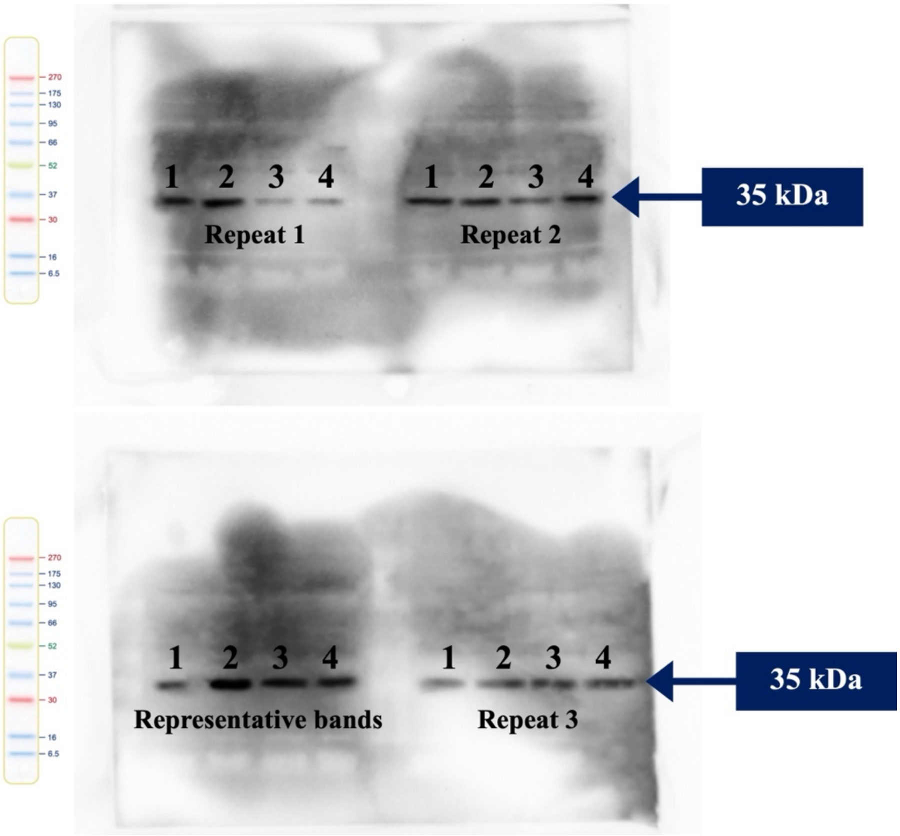

Figure S5: Western blotting was used to detect the expression of caspase-3 in response to the effects of phytosome-encapsulated 6-gingerol and 6-shogaol-enriched extracts from *Zingiber officinale* Roscoe (6GS) on hydrogen peroxide-induced neurotoxicity in SH-SY5Y cells. The groups included: (1) control, (2) hydrogen peroxide + vehicle, (3) hydrogen peroxide + 6GS at a dose of 15.625 μg/mL, and (4) hydrogen peroxide + 6GS at a dose of 31.25 μg/mL.

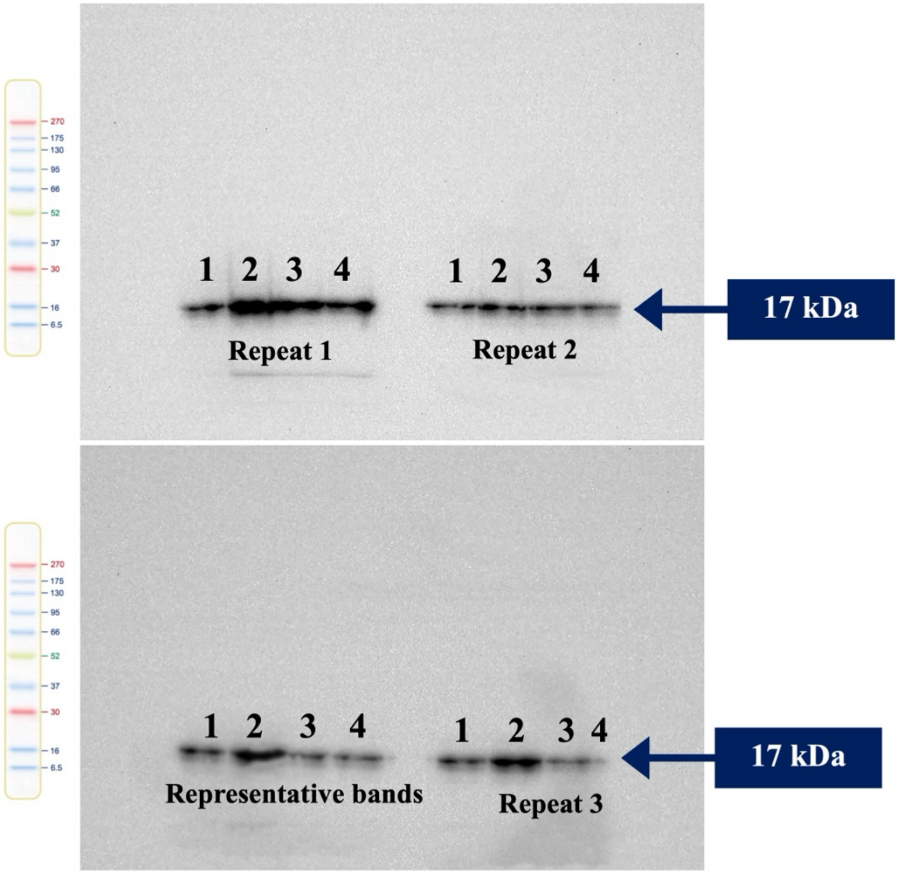

Figure S6: Western blotting was used to detect the expression of TNF-α in response to the effects phytosome-encapsulated 6-gingerol and 6-shogaol-enriched extracts from *Zingiber officinale* Roscoe (6GS) on hydrogen peroxide-induced neurotoxicity in SH-SY5Y cells. The groups included: (1) control, (2) hydrogen peroxide + vehicle, (3) hydrogen peroxide + 6GS at a dose of 15.625 μg/mL, and (4) hydrogen peroxide + 6GS at a dose of 31.25 μg/mL.
